# Assessment of Micro- and Macronutrient Intake in Male Competitive Athletes Using the Epic-Norfolk Food Frequency Questionnaire

**DOI:** 10.3390/life15030458

**Published:** 2025-03-13

**Authors:** Ana Stupin, Leon Perić, Ivana Jukić, Alina Boris, Lorena Stanojević, Ines Drenjančević

**Affiliations:** 1 Department of Physiology and Immunology, Faculty of Medicine Osijek, Josip Juraj Strossmayer University of Osijek, 31000 Osijek, Croatia; ana.stupin@mefos.hr (A.S.); lperic1@yahoo.com (L.P.); ivana.jukic@mefos.hr (I.J.); 2 Scientific Centre of Excellence for Personalized Health Care, Josip Juraj Strossmayer University of Osijek, 31000 Osijek, Croatia; 3 Department of Ophthalmology, University Hospital Center Osijek, 31000 Osijek, Croatia; 4 Faculty of Medicine Osijek, Josip Juraj Strossmayer University of Osijek, 31000 Osijek, Croatia; aboris@mefos.hr (A.B.); lstanojevic@mefos.hr (L.S.)

**Keywords:** athletes, nutrition, macronutrients, micronutrients, food frequency questionnaire, sodium-to-potassium ratio, vitamin D, folate

## Abstract

This study aimed to investigate diet quality in a population of male competitive athletes by comparing their total energy and macro- and micronutrients intake assessed by a food frequency questionnaire (FFQ) to current nutritional recommendations for this specific population. An additional goal was to assess athletes’ sodium-to-potassium (Na-to-K) ratio. This cross-sectional study involved 31 healthy competitive male athletes. Subjects’ body mass index, body composition, arterial blood pressure, as well as red blood count (RBC) and serum lipid profile measurements showed that all subjects were lean and normotensive and had normal RBC and serum lipid levels. All subjects completed a cross-culturally adapted, translated, and validated EPIC–Norfolk FFQ. The total energy intake reported in the FFQ was below the recommended values; however, 71% of subjects were classified as low energy reporters (LERs). Energy intake from proteins was within and from carbohydrates was below the recommended interval, while the total energy intake from fats (and also saturated fatty acids) exceeded the recommendations. Suboptimal intakes of vitamin D and folate were observed, indicating the necessity for monitoring (and supplementing) in this population. High Na intake, which despite adequate K intake resulted in a higher Na-to-K ratio, highlighted concerns over excessive salt consumption, but also accentuated the need for monitoring sodium (and potassium) intake in competitive athletes with consideration for sweat-related losses.

## 1. Introduction

Proper nutrition, although with specific recommendations for different types of sports, is essential for all athletes (e.g., endurance, strength, and mixed-sport athletes) to support their physical activity, attain optimal physical condition, improve athletic performance, and aid in recovery after exercise [1]. To meet their nutritional needs and maximize performance, athletes must carefully monitor their diet, paying attention to the timing, quantity, and nutritional quality of their food intake, as well as ensure proper hydration [1,2]. A combination of key nutrients including carbohydrates, proteins, fats, vitamins, and minerals play a crucial role in athlete’s nutrition, as it serves as an energy source and unavoidable factor for success in sports. Athletes who fail to consume sufficient energy and fail to maintain a balanced intake of both macronutrients and micronutrients may face challenges in training adaptation and recovery [1,2]. Specifically, insufficient energy intake can lead to decreased lean mass, immune function impairment, reduced bone mineral density, a heightened risk of injury, and a greater occurrence of symptoms linked to overtraining [1,2].

Among macronutrients, carbohydrates are especially important for athletic performance, recovery, and replenishing glycogen stores for future training sessions, as muscle glycogen and blood glucose serve as the primary energy sources for active muscles. However, athletes often fail to meet the recommended carbohydrate intake, whose needs may vary depending on training intensity between 5 and 12 g/kg of body weight [1,2]. Changes in muscle protein synthesis rates are key factors influencing muscle mass changes over time, and both exercise and diet play significant roles in them [3]. For both endurance and strength athletes, the primary goal of protein intake is to repair and restore skeletal muscle and connective tissue [4]. The amount, timing, and type of protein consumed can impact the extent of muscle remodeling following different types of exercise. Consuming protein before, during, and after exercise has been shown to have a positive effect on muscle protein synthesis [5]. The recommended protein intake for endurance athletes is between 1.2 and 2.0 g/kg of body weight per day [1,4]. During the period of 1985–2005, a number of studies investigated effects of high-fat diets in athletes, particularly aimed at increasing intramuscular triglyceride stores. Nowadays, there is consensus that adequate fat intake is important, but there is also an ongoing debate whether high-fat or fat-loading diets are effective in enhancing performance in different types of trained individuals [6,7,8]. Athletes are recommended to consume between 20 and 35% of their total calorie intake from fat, with less than 10% of that coming from saturated fat [6].

Similar to the impact on macronutrient needs, exercise intensity, duration, and character also influence athletes’ micronutrient requirements. The most important micronutrients for athletes are iron, calcium, vitamin D, magnesium, zinc, vitamin C, B vitamins, sodium, and potassium [1]. Sports with higher energy demands typically correspond to higher micronutrient requirements, although the precise quantification remains challenging [9]. While a balanced diet can usually meet the recommended reference intakes for vitamins and minerals, substantial losses through sweat, urine, or certain dietary habits can result in an increased demand for these nutrients [9]. Athletes may opt for external supplementation to enhance their well-being and performance, particularly for winter sports, indoor activities, or in conditions such as altitude training [10,11].

High sodium intake and inadequate potassium consumption, both common in Western diets, are key dietary factors linked to arterial hypertension and cardiovascular diseases [12]. In recent years, the combined effect of sodium and potassium on cardiovascular health has led to the introduction of the sodium-to-potassium ratio (Na-to-K) as a more reliable measure of cardiovascular risk and related mortality than sodium or potassium intake alone [13,14]. The World Health Organization (WHO) recommends a daily intake of less than 2000 mg of sodium and more than 3510 mg of potassium, aiming for an optimal Na-to-K ratio of less than 1.0 to maintain cardiovascular health [15]. However, due to low adherence to these guidelines and evidence suggesting that Na-to-K ratios between 1.0 and 2.0 can still help lower the risk of cardiovascular disease, a Na-to-K ratio below 2.0 was suggested as a more achievable target [16]. Although salt needs in athletes may be higher than in the general population due to the increased loss of electrolytes through sweat during intense physical activity, the intake of Na and K should be considered in composing the athlete’s diet, too.

To optimize athletes’ performance and overall well-being, while reducing the risk of potential nutritional imbalances, it is essential to tailor their diet according to established guidelines. The hypothesis of the present study is that athletes, despite widely accessible recommendations on nutrients intake correspondent to their specific needs, do not have a balanced diet. This study aimed to assess the diet quality of male competitive endurance athletes by comparing their total energy, macronutrient, and micronutrient intake from a food frequency questionnaire to the current nutritional recommendations for this population given by the European Food Safety Authority (EFSA) [17]. An additional goal was to evaluate the athletes’ sodium-to-potassium (Na-to-K) ratio and identify potential factors influencing this ratio within the observed group.

## 2. Materials and Methods

### 2.1. Study Population

This was a cross-sectional study involving 31 voluntary healthy, competitive male athletes. Participants were recruited from local track and field clubs between May 2023 and April 2024. All athletes had been actively training for at least 12 months, with a minimum of 5 training sessions per week, primarily focused on endurance (middle-distance and long-distance runners). Eligibility criteria included being between the ages of 18 and 45 and having a body mass index (BMI) between 18.5 and 29.99 kg/m^2^. Exclusion criteria consisted of smoking, hypertension, hyperlipidemia, diabetes, coronary disease, kidney damage, cerebrovascular or peripheral artery diseases, or any other condition that could affect vascular and endothelial function.

Before participating, each individual was fully informed about the study protocol and procedures, and all provided written informed consent. Measurements, sampling, and completion of the food frequency questionnaire (FFQ) were carried out during a single study visit scheduled in morning hours after an overnight fasting. The study adhered to the latest revision of the Declaration of Helsinki and was approved by the Ethics Committee of the Faculty of Medicine Osijek (Cl: 602-04/23-08/03; No: 2158-61-46-23-89). This study was part of a clinical trial examining the effects of carnosine supplementation on cardiovascular function, registered at ClinicalTrials.gov (NCT05723939 Carnosine Supplementation and Cardiovascular Function).

### 2.2. Subjects Characteristics

Participants’ height and weight were measured using a RADWAG scale (RADWAG, Toruńska 5, 26-600 Radom, Poland) to calculate their BMI. Participants’ waist and hip circumferences were measured with a tape measure to calculate the waist-to-hip ratio (WHR). The final arterial blood pressure value was determined by averaging three consecutive measurements taken with an Microlife BP B3 AFIB device (Microlife AG Swiss Corporation, Espenstrasse 139, 9443 Widnau, Switzerland).

Fasting venous blood samples were collected using BD collection tubes (BD Becton, Dickinson and Company, Franklin Lakes, NJ, USA) and analyzed for red blood cell count (RBC) and lipid profile (including total cholesterol, triglycerides, LDL cholesterol, and HDL cholesterol) at the Department of Clinical Laboratory Diagnostics, Osijek University Hospital, Osijek, Croatia.

### 2.3. Body Composition and Body Fluid Status

Body composition and body fluid statuses were measured by a 4-terminal portable bioelectrical impedance analyzer (Maltron Bioscan 920-II, Maltron International Ltd., 20 Sirdar Rd, Rayleigh SS6 7XF, Essex, UK) (impedance accuracy: ±3R across 5–1100 range; phase accuracy: ±0.1 degree). The analysis was performed in a supine position, subjects having arms by their side and separated from the trunk, and legs separated from each other. Four sensing electrodes placed on the dorsum of the wrist and the anterior surface of the ankle were used for whole-body measurements. The manufacturer’s original software using empirically derived formulas generated data on the proportion of muscle mass, fat free mass, fat mass, total body water, extracellular water (ECW), intracellular water (ICW), plasma volume, interstitial fluid volume, and body density.

### 2.4. Food Frequency Questionnaire (FFQ)

The publicly available EPIC–Norfolk food frequency questionnaire (FFQ) (https://www.epic-norfolk.org.uk/wp-content/uploads/2020/11/CAMB-PQ-6-1205a_front.pdf, accessed on 10 February 2022) was used to assess each participant’s average dietary intake over the past year. The EPIC-Norfolk FFQ was previously downloaded, translated into Croatian, cross-culturally adapted, and validated [18]. The validation process revealed that the Croatian version of the EPIC–Norfolk FFQ is a useful tool for assessing dietary intakes in young people in Croatia and possibly in neighboring countries with similar languages and dietary habits [18]. The questionnaire is divided into two sections. Part 1 contains a food list with 130 items, each associated with a portion size. Participants were asked to select the frequency of consumption for each food item from nine available categories. The food list was slightly adjusted to reflect brands available in the Croatian market. Part 2 includes additional questions, some of which provide more detailed information related to the food items in Part 1.

The data collected from the EPIC–Norfolk FFQ were entered into a spreadsheet following the provided guidelines and processed using the FFQ EPIC Tool for Analysis (FETA; FETA tool is based on version 6 of the EPIC-Norfolk FFQ.) (https://www.epic-norfolk.org.uk/for-researchers/ffq/, accessed on 2 December 2024). FETA is a freely available, stand-alone tool that calculates nutrient and food group intake from EPIC–Norfolk FFQ data. It generates nutrient output that includes the average daily intake of 46 nutrients and 14 basic food groups [19,20]. The FFQ also supports supplementation intake (vitamins, minerals, fish oils, fibers, or other food supplements).

To evaluate reported energy intake at the individual level, Goldberg cut-offs were used [21]. The ratio between reported energy intake (EIrep) and resting metabolic rates (RMR) (EIrep/RMR) was calculated for each participant. Age specific PAL values for very active physical activity adopted by the EFSA NDA Panel was applied to determine cut-offs to establish misreporting of energy intake for individuals aged 15–69 years (PAL = 2.0). The calculated cut-offs for this activity level and age range (18–69 years) were a lower cut-off of 1.120 and an upper cut-off of 2.892 [22]. Each participant’s EIrep/RMR was compared with these cut-offs, categorizing them as either low-energy reporters (LERs) or non-LERs.

### 2.5. Statistical Analysis

All data were presented as the arithmetic mean and standard deviation (SD). The Shapiro–Wilk test was applied to assess the normality of data distribution. Mean values from the FFQ that deviated by more than 10% from the recommended average requirement (AR) or population reference intake (PRI) were considered significantly different. Pearson’s correlation test was used to examine correlations between normally distributed variables, while Spearman’s correlation test was applied for non-normally distributed variables. A multiple linear regression analysis was conducted to determine which food group intakes most significantly influenced macronutrients and micronutrients in the study population. Due to the large number of correlated variables, only significant correlations were presented in the Results Section. A *p*-value of less than 0.05 was considered statistically significant. Statistical analyses were performed using SigmaPlot, version 11.2 (Systat Software, Inc., Chicago, IL, USA).

## 3. Results

Subjects’ general characteristics, arterial blood pressure, red blood count, and serum lipid profiles are presented in Table 1. They were all endurance athletes, were lean and normotensive, and had normal RBC and serum lipid levels.

In addition, a body composition and body fluid status analysis demonstrated that all subjects had adequate muscle mass, as it was higher than 20 kg, which is considered the limit value when defining low muscle mass. Also, the distribution between lean and fat mass, and also total body water, was adequate. The body composition and body fluid status analysis results are presented in Table 2.

The FFQ was completed by all subjects, and all were included in the statistical analysis. The daily energy and macronutrient intake of male competitive athletes is shown in Table 3. The reported energy intake (EIrep) in the athletes was similar to the calculated resting metabolic rate (RMR) (EIrep 2105 ± 666 vs. RMR 1972 ± 146, *p* = 0.871) but was significantly lower than the recommended daily energy intake (3350 kcal/day) for this specific population. The further calculation of a mean EIrep/RMR ratio, which was 1.07 ± 0.35, indicated that, according to Goldberg cut-offs, 71% of subjects were low-energy reporters (LERs). The protein-derived energy expressed as the percent of total energy (19.9%) was within the recommended interval (10–35% of energy), as well when expressed as daily intake in grams per kg of body weight (FFQ protein intake, 1.3 ± 0.5 g/kg BW; recommended protein intake, 1.2–2.0 g/kg BW) [1]. The carbohydrate intake expressed as intake in grams per kg of body weight was substantially lower than the recommendations (FFQ carbohydrates intake, 2.8 ± 1.1 g/kg BW; recommended carbohydrates intake, 5–12 g/kg BW) [1]. The total fat intake (38.0%) was slightly above the recommended reference value (20–35% of energy). In addition, the fat intake in the form of saturated fatty acids accounted for 14.4 ± 2.4% of the total energy intake, which was above the recommended 10% [1]. There was no significant correlation between blood lipids level (total cholesterol, triglycerides, HDL, and LDL cholesterol) and daily macronutrient intake (*p* > 0.05).

The daily intake of vitamins and micronutrients in male competitive athletes, as determined by the EPIC–Norfolk FFQ, is shown in Table 4. Data collected using the FFQ were compared with the average requirement (AR) or population reference intake (PRI) reported for a given age, sex group, and level of physical activity (PAI) (https://multimedia.efsa.europa.eu/drvs/index.htm, n.d., accessed on 2 December 2024), with a deviation of more than 10% considered suboptimal or excessive. Compared to the AR or PRI, the FFQ results showed a suboptimal daily intake of vitamin D, folate, and copper. The daily intake of vitamin A, vitamin E, vitamin C, potassium, calcium, iron, magnesium, manganese, selenium, and zinc were found to be optimal, while the daily intake of niacin, riboflavin, thiamine, vitamin B6 and vitamin B12, sodium, chloride, and iodine was above the AR or PRI values (i.e., excessive).

The daily intake in grams determined by the FFQ was used to calculate the molar Na-to-K ratio, which was 1.53 ± 0.36. The estimated daily salt intake (NaCl) in competitive male athletes based in the FFQ results was 8.11 ± 2.80 g per day, which was above the WHO recommended values of 5 g/day. There was no significant correlation between daily sodium or potassium intake and arterial blood pressure level (systolic, diastolic, and mean arterial blood pressure) (*p* > 0.05).

The daily food group intakes in competitive male athletes are presented in Table 5. According to a multiple linear regression analysis, the daily protein intake was mostly derived from cereals and cereal products (*p* = 0.042), eggs and egg dishes (*p* = 0.014), and meat and meat products (*p* < 0.001). Carbohydrate daily intakes were mostly derived from the intake of cereals and cereal products (*p* < 0.001), fruits (*p* < 0.001), and potatoes (*p* < 0.001), while fat intake was derived from fruit (*p* = 0.040), meat and meat products (*p* < 0.001), nuts and seeds (*p* < 0.001), soups and sauces (*p* = 0.005), and sugars and snacks (*p* < 0.001). Daily sodium (and iodine) intake was significantly derived from cereals and cereal products (*p* < 0.001), nuts and seeds (*p* < 0.001), potatoes (*p* = 0.001), and soups and sauces (*p* < 0.001).

## 4. Discussion

The total energy intake of healthy competitive male athletes reported in the FFQ was below the recommended values; however, according to the Goldberg cut-off analysis, 71% of the subjects were classified as low-energy reporters (LERs). Moreover, athlete muscle mass and fat tissue percentages were within the given reference intervals for athletes, indicating that they were not in a chronic energy deficit. The energy intake from proteins was within the recommended interval, the carbohydrate intake was lower than the recommendations, while the total energy intake from fats exceeded the recommended guideline. Moreover, the intake of saturated fatty acids was higher than the recommendation. While athletes had an optimal intake of most micronutrients and vitamins, a suboptimal intake of vitamin D and folate was observed, which play an important role in athletes; bone health, skeletal muscle growth, and a high red blood cell turnover rate depend on these nutrients. In addition, sodium and iodine intakes were above recommended levels, once again highlighting the general problem of excessive salt intake, which is associated with increased cardiovascular risk at the population level.

### 4.1. Energy Intake in Competitive Athletes

The results of this study suggest a noticeable negative energy balance in competitive athletes (an average of 1245 kcal/day), which aligns with findings from similar research on energy intake in athletes. Previous studies have reported negative energy balances during the preparation phase in male cross-country skiers [24], and male runners [25], averaging 304 kcal/day for men athletes [26]. For male cyclists [27,28], triathletes [29], and runners [30], the negative energy balance during the competition phase was even more pronounced, averaging 2177 kcal/day. The most probable cause of these energy deficits is the frequent issue of underreporting energy intake through self-assessment in human studies. A review of nine studies using doubly labeled water (DLW) to validate self-reported energy intake in athletes found that underreporting can account for 10–45% of total energy expenditure, with the discrepancy growing as energy requirements increase [31]. Even after increasing athletes’ reported energy intake by 45%, negative energy balances were still observed during both the preparation and competition phases [31]. Another potential reason for the negative energy balance could be the limited accuracy and precision of the methods used to estimate energy intake in athletes. Although short-term prospective dietary records (ideally 3–7 days) are considered more accurate than food frequency questionnaires (FFQs) and dietary recalls, as these methods rely on memory and tend to be less precise, error rates remain high even when dietary records are used. Despite being regarded as the “gold standard” for dietary assessment, dietary records place a significant burden on participants to document their food intake accurately and truthfully, and researchers are dependent on appropriate databases to code data correctly [31,32,33,34]. Furthermore, athlete cohorts face additional challenges, such as the difficulty of recording large amounts of food, frequent eating occasions, irregular meal patterns, estimating large portion sizes, and accounting for sports nutrition and supplements [31]. Several recent systematic reviews have raised concerns about the reliability of both types of self-reported food intake data, suggesting that they should not be relied upon as an accurate measure of energy intake [35]. One limitation of the present study was the use of the FFQ to assess energy intake, and the fact that we did not calculate total energy expenditure for each participant. Instead, we used the average predicted energy expenditure for competitive male athletes based on age and sport. Despite this, the results aligned with those of previous studies, confirming that athletes consistently underestimate their energy intake across nearly all available methods. What distinguishes this study from others was the use of Goldberg cut-offs to evaluate reported energy intake on an individual level using the FFQ, which revealed that nearly 71% of the athletes were classified as low-energy reporters. Our findings underscore the urgent need for improved methods to quantify dietary intake. especially in athletes, such as the use of nutritional biomarkers and automated image analyses of food and beverage consumption [35].

### 4.2. Macronutrient Intake in Competitive Athletes

The findings on substantially lower daily carbohydrate intake in competitive athletes are consistent with previous studies, showing that competitive athletes do not consume the recommended amounts of carbohydrates to maximize energy supply for training and recovery processes. A recent systematic review of dietary intake in elite athletes in twenty studies found that carbohydrate intake in men ranged from 221 to 350 g/day, 40–61%E and 3.0 to 5.3 g/kg body weight/day [36], which is very similar to the present results. These results suggest the need for targeted education in this highly physically active population to optimize carbohydrate intake, which is particularly important given the current negative messages about carbohydrates all over the public and social media. In terms of macronutrients, protein intake among male athletes was within the recommended range, but at the lower end of the recommendations. This was also consistent with a recent systematic review that included 12 studies reporting that protein intake in younger male athletes ranged from 1.0 g/kg body weight/day to 2.0 g/kg body weight/day [36]. Although protein intake in male athletes appeared to be adequate, it should be emphasized that protein intake was close to the lower limit of recommendations. Additionally, protein intake should be prioritized in athletes in order to stimulate muscle protein synthesis and minimize lean tissue loss. The fat intake of young athletes exceeded the recommended values, which was also consistent with previously published studies. A systematic review of 18 studies found that the average daily fat intake in male athletes ranged from 51 to 134 g/day, 22 to 41%E and 1.0 to 9.0 g/kg body weight/day [36]. One study found that younger male athletes consumed less saturated fat compared to inactive controls, while in the current study, the proportion of saturated fat exceeded the recommended 10%. The suboptimal carbohydrate intake and high fat intake observed in the present study may reflect the popularity of low-carbohydrate/high-fat diets among athletes, which are claimed to induce ‘glycogen sparing’ to enhance performance [37], but this is beyond the scope of the present study.

### 4.3. Vitamin D Intake in Competitive Athletes

Even though in addition to dietary intake, vitamin D may be synthesized in the skin during exposure to sunlight, a significant proportion of athletes, especially in Europe, experience vitamin D deficiency [38]. For example, studies have shown that vitamin D levels in athletes across various sports, including running, basketball, and gymnastics, are comparable to those in the general population, with deficiency levels varying based on factors like geographical location and whether the sport is indoors or outdoors [39]. This deficiency has been linked to impaired strength and endurance [40], as well as an increased risk of injury [41,42]. Even though we did not measure vitamin D serum concentrations, which could be considered as a study limitation, the present study supports earlier findings, showing inadequate dietary vitamin D intake among male athletes, which emphasizes the need to assess and supplement vitamin D levels, especially during the winter months. Vitamin D supplementation can boost serum 25(OH)D levels, enhance strength, reduce injury rates, and improve physical performance [43,44,45]. Thus, it is important to regularly monitor vitamin D levels in athletes to ensure optimal health and athletic performance [46].

### 4.4. Folic Acid Intake in Competitive Athletes

Recent national dietary surveys of European populations have shown that very few countries meet the recommended daily allowance (RDA) of 400 μg for folic acid. The average intake of folic acid was 268 μg (ranging from 129 to 399 μg) for women and 318 μg (ranging from 142 to 643 μg) for men. Only young and older Irish men, as well as middle-aged Lithuanian and Turkish men, had adequate folic acid intake [47]. Additionally, insufficient folic acid intake has been observed in athletes across various sports [48,49], which aligns with the findings of the present study. Folic acid, an essential vitamin, may enhance nitric-oxide-dependent vasodilation in the skin microvasculature of older adults through both local administration and chronic ingestion. This effect might be due to a direct interaction with endothelial nitric oxide synthase or the restoration of tetrahydrobiopterin (BH4) availability [50,51]. Folic acid has also been shown to improve macrovascular function in young female athletes with vascular dysfunction [52]. Therefore, folic acid supplementation could potentially serve as a therapy to enhance nitric-oxide-dependent vasodilation and improve muscle perfusion in adults. However, no data on the effect of folic acid for male athletes are available at the moment.

### 4.5. Sodium, Potassium, and Na/K Ratio in Competitive Athletes

Recent findings from a national study on the consumption of salt, potassium, and iodine in the general adult population of Croatia revealed an average daily salt intake of 8.6 g/day (10.5 g for men and 8.0 g for women) [53], which still exceeds the WHO’s recommended limit of 5 g/day. As stated earlier, although salt needs in athletes may be higher than in the general population due to the increased loss of electrolytes through sweat during intense physical activity, there are no specific dietary recommendations on Na and K intake specifically for the athlete population. The present study indicated that the primary sources of dietary sodium for athletes were cereals and cereal products, nuts and seeds, potatoes, and soups and sauces, highlighting food groups that should be monitored to reduce salt intake in both athletes and the general population. The average potassium intake in the Croatian adult population was 2.9 g/day [53], which was notably below the WHO target of >3.5 g/day but higher than the global average of 2.3 g/day [54]. The current study found that, contrary to the general Croatian population, competitive male athletes had a significantly higher potassium intake (3.6 ± 1.2 g/day) and importantly, their intake was within the WHO’s recommended range. Regarding iodine, most participants in the Croatian national study (EH-UH2 study) had urinary iodine excretion categorized as “adequate” or “above requirement”, indicating that the overall median iodine levels were within the recommended range [53], which was also true for male competitive athletes in this study. It is important to emphasize that the daily salt intake in athletes was not associated with arterial blood pressure level, which was consistent with earlier studies and confirms blood pressure salt-resistance in younger individuals but does not exclude high-salt BP-independent adverse effects on cardiovascular health (e.g., vascular and endothelial function) [55]. Taken together, the findings show that male athletes consume less table salt (8.1 g/day on average) compared to the Croatian general male population, albeit still above recommended values. More importantly, their daily potassium intake was significantly higher than that of the average Croatian male population and met the WHO guidelines. These results suggest that male athletes have a better diet than the general population in terms of lower salt intake and higher potassium intake, reflecting their awareness of the importance of reducing table salt and increasing potassium intake to maintain cardiovascular health. However, because of electrolyte sweat loss, the precise determination of the Na-to-K ratio in endurance athletes may require 24-urine sampling for estimating sodium and potassium excretion.

## 5. Conclusions

In conclusion, the FFQ analysis of healthy competitive male athletes showed a generally optimal protein but low carbohydrate intake, while their total energy intake was below recommended levels, with many classified as low-energy reporters. Fat intake exceeded guidelines, especially saturated fats. Suboptimal levels of vitamin D and folate were observed, indicating the necessity for vitamin D and folate monitoring (and supplementing) in this population. Additionally, sodium intake was higher than recommended, which despite adequate potassium intake, resulted in a high sodium-to-potassium ratio, highlighting concerns over excessive salt consumption. An individual approach to a balanced diet depending on the training process and location, exercise intensity, and sweating and hydration should always be the ultimate goal in meeting the needs of this nutritionally demanding population.

## Figures and Tables

**Table 1:** General characteristics, arterial blood pressure, red blood count, and lipid profiles of male competitive endurance athletes.

Variable	Subjects	Reference Values *
Number	31	
Age (year)	24 ± 6	
Body mass index (kg/m^2^)	24.5 ± 2.2	18.5–24.9
Waist-to-hip ratio	0.82 ± 0.04	<0.95
Systolic blood pressure (mmHg)	132 ± 10	<140
Diastolic blood pressure (mmHg)	77 ± 7	<90
Mean arterial pressure (mmHg)	95 ± 7	70–100
Erythrocytes (10 × 10^12^/L)	5.12 ± 0.30	4.34–5.72
Hemoglobin (g/L)	148 ± 8	138–175
Hematocrit (%)	0.440 ± 0.020	0.415–0.530
MCV (fL)	86.8 ± 3.3	83.0–97.2
MCH (pg)	28.8 ± 1.1	27.4–33.9
MCHC (g/L)	332 ± 8	320–345
RDW-CV (%)	12.6 ± 0.5	9.0–15.0
Cholesterol (mmol/L)	4.31 ± 0.79	<5.00
Triglycerides (mmol/L)	0.89 ± 0.55	<1.70
HDL cholesterol (mmol/L)	1.46 ± 0.27	<1.00
LDL cholesterol (mmol/L)	2.82 ± 0.62	<3.00

Results are presented as average and standard deviation (SD). * Department of Clinical Laboratory Diagnostics, Osijek University Hospital, Osijek, Croatia. MCH- mean corpuscular hemoglobin; MCHC- mean corpuscular hemoglobin concentration; RDW-CV- red cell distribution width; HDL- high-density lipoprotein; LDL- low-density lipoprotein.

**Table 2:** Body composition and body fluid status of male competitive athletes.

Variable	Subjects	Reference Values [23]
Muscle mass (kg)	37.0 ± 10.5	<20 kg low muscle mass
Fat free mass (%)	84.5 ± 4.7	80–90%; <75% obesity
Fat (%)	15.5 ± 4.7	10–20%; >25% obesity
Total body water (%)	66.1 ± 7.5	55–70%
Extracellular water (%)	49.1 ± 9.4	
Intracellular water (%)	50.9 ± 9.4	
ECW/ICW	1.04 ± 0.46	
Plasma fluid (L)	5.6 ± 1.4	
Interstitial fluid (L)	19.7 ± 4.8	
Body density (kg/L)	1.05 ± 0.02	

ECW—extracellular water; ICW—intracellular water.

**Table 3:** Daily energy and macronutrient intake in competitive male athletes assessed by EPIC–Norfolk food frequency questionnaire (FFQ).

	FFQ	AR or PRI *
Reported energy intake (EIrep) (kcal/day)	2105 ± 666	3350
Resting metabolic rate (RMR) (kcal) †	1972 ± 146	
EIrep/RMR	1.07 ± 0.35	
LER, number (%)	22 (71.0%)	
nonLER, number (%)	9 (29.0%)	
Protein (g/day)	105 ± 43	
Protein (g/kg BW)	1.25 ± 0.47	1.2–2.0 g/kg BW
Protein (%E)	19.9 ± 4.3	10–35% of energy
Carbohydrates (g/day)	231 ± 86	
Carbohydrates (g/kg BW)	2.8 ± 1.1	5–12 g/kg BW
Carbohydrates (%E)	43.9 ± 6.1	45–60% of energy
Fats (g/day)	89 ± 30	
Fats (%E)	38.0 ± 3.7	20–35% of energy

Results are presented as average and standard deviation (SD). AR—average requirement; PRI—population reference intake; LER—low-energy reporter; BW—body weight. * AR and PRI are given as requirements of specified age and sex group and for specified level of physical activity (PAI 2.0), which reflects very active lifestyle (https://multimedia.efsa.europa.eu/drvs/index.htm, accessed on 2 December 2024). † RMR was calculated using the Harris and Benedict equation.

**Table 4:** Daily vitamin and micronutrient intake assessed by EPIC–Norfolk food frequency questionnaire (FFQ) in competitive male athletes.

Nutrient	FFQ	AR or PRI *	FFQ vs. AR or PRI **
Vitamin A (μg/day)	678 ± 484	750	↔
Vitamin D (μg/day)	3.6 ± 2.2	15	↓
Vitamin E (mg/day)	11.8 ± 5.0	13	↔
Folate (μg/day)	268 ± 101	330	↓
Niacin (mg/day)	27 ± 11	18	↑
Riboflavin (mg/day)	2.1 ± 0.8	1.6	↑
Thiamin (mg/day)	1.7 ± 0.6	1.4	↑
Vitamin B6 (mg/day)	2.5 ± 0.9	1.7	↑
Vitamin B12 (μg/day)	7.8 ± 4.8	4	↑
Vitamin C (mg/day)	109 ± 86	110	↔
Sodium (mg/day)	3192 ± 1103	2000	↑
Potassium (mg/day)	3624 ± 1199	3500	↔
Calcium (mg/day)	991 ± 368	950	↔
Chloride (g/day)	4.7 ± 1.6	3.1	↑
Copper (mg/day)	1.3 ± 0.5	1.6	↓
Iron (mg/day)	11.2 ± 4.0	11	↔
Iodine (μg/day)	168 ± 64	150	↑
Magnesium (mg/day)	322 ± 97	350	↔
Manganese (mg/day)	2.7 ± 0.9	3	↔
Selenium (μg/day)	73 ± 30	70	↔
Zinc (mg/day)	11.9 ± 5.3	11	↔

Results are presented as average and standard deviation (SD). * AR—average requirement; PRI—population reference intake. ** Variation > 10%.

**Table 5:** Food group intakes in competitive male athletes assessed by EPIC–Norfolk food frequency questionnaire (FFQ).

	FFQ
Alcoholic beverages (g/day)	44.7 ± 51.8
Cereals and cereal products (g/day)	277 ± 112
Eggs and egg dishes (g/day)	50 ± 57
Fats and oils (g/day)	12 ± 6
Fish and fish products (g/day)	33 ± 40
Fruit (g/day)	286 ± 284
Meat and meat products (g/day)	195 ± 120
Milk and milk products (g/day)	385 ± 218
Non-alcoholic beverages (g/day)	342 ± 361
Nuts and seeds (g/day)	11 ± 11
Potatoes (g/day)	80 ± 54
Soups and sauces (g/day)	138 ± 194
Sugars (g/day)	43 ± 63
Vegetables (g/day)	184 ± 135

Results are presented as average and standard deviation (SD). AR—average requirement; PRI—population reference intake. AR and PRI are given as requirements of specified age and sex group and for specified level of physical activity (PAI 2.0), which reflects a very active lifestyle.

## Data Availability

Original data will be shared upon reasonable request to the corresponding author.

## Abbreviations

The following abbreviations are used in this manuscript:

FFQ	Food frequency questionnaire
Na-to-K	Sodium-to-potassium ratio
RBC	Red blood count
LERs	Low-energy reporters
WHO	World Health Organization
EFSA	European Food Safety Authority
BMI	Body mass index
WHR	Waist-to-hip ratio
ECW	Extracellular water
ICW	Intracellular water
FETA	FFQ EPIC Tool for Analysis
EIrep	Reported energy intake
RMR	Resting metabolic rate
LER	Low-energy reporters
SD	Standard deviation
AR	Average requirement
PRI	Population reference intake
BW	Body weight
RDA	Recommended daily allowance
BH4	Tetrahydrobiopterin
